# Determinants of Crimean–Congo haemorrhagic fever virus exposure dynamics in Mediterranean environments

**DOI:** 10.1111/tbed.14720

**Published:** 2022-10-17

**Authors:** Raúl Cuadrado‐Matías, Sara Baz‐Flores, Alfonso Peralbo‐Moreno, Gloria Herrero‐García, María A. Risalde, Patricia Barroso, Saúl Jiménez‐Ruiz, Carmen Ruiz‐Rodriguez, Francisco Ruiz‐Fons

**Affiliations:** ^1^ Instituto de Investigación en Recursos Cinegéticos (IREC) CSIC‐UCLM‐JCCM Ciudad Real Spain; ^2^ Departamento de Sanidad Animal Facultad de Veterinaria Universidad de León León Spain; ^3^ Departamento de Anatomía y Anatomía Patológica Comparadas y Toxicología, Grupo de Investigación en Sanidad Animal y Zoonosis (GISAZ), UIC Zoonosis y Enfermedades Emergentes ENZOEM Universidad de Córdoba Córdoba Spain; ^4^ CIBERINFEC (ISCIII), CIBER de Enfermedades Infecciosas Instituto de Salud Carlos III Madrid Spain; ^5^ Grupo de Investigación en Sanidad Animal y Zoonosis (GISAZ), Departamento de Sanidad Animal Universidad de Córdoba Córdoba Spain

**Keywords:** disease ecology, host–tick–pathogen interactions, tick, wild ungulates, zoonosis

## Abstract

Crimean–Congo haemorrhagic fever (CCHF) is an emerging tick‐borne human disease in Spain. Understanding the spatiotemporal dynamics and exposure risk determinants of CCHF virus (CCHFV) in animal models is essential to predict the time and areas of highest transmission risk. With this goal, we designed a longitudinal survey of two wild ungulate species, the red deer (*Cervus elaphus*) and the Eurasian wild boar (*Sus scrofa*), in Doñana National Park, a protected Mediterranean biodiversity hotspot with high ungulate and CCHFV vector abundance, and which is also one of the main stopover sites for migratory birds between Africa and western Europe. Both ungulates are hosts to the principal CCHFV vector in Spain, *Hyalomma lusitanicum*. We sampled wild ungulates annually from 2005 to 2020 and analysed the frequency of exposure to CCHFV by a double‐antigen ELISA. The annual exposure risk was modelled as a function of environmental traits in an approach to understanding exposure risk determinants that allow us to predict the most likely places and years for CCHFV transmission. The main findings show that *H. lusitanicum* abundance is a fundamental driver of the fine‐scale spatial CCHFV transmission risk, while inter‐annual risk variation is conditioned by virus/vector hosts, host community structure and weather variations. The most relevant conclusion of the study is that the emergence of CCHF in Spain might be associated with recent wild ungulate population changes promoting higher vector abundance. This work provides relevant insights into the transmission dynamics of CCHFV in enzootic scenarios that would allow deepening the understanding of the ecology of CCHFV and its major determinants.

## 1 INTRODUCTION

Crimean–Congo haemorrhagic fever (CCHF) is one of the human viral diseases of greatest concern to the World Health Organization because of its high lethality, the absence of efficient prophylactic measures (Keshtkar‐Jahromi et al., 2011), its capacity for human‐to‐human transmission (Tsergouli et al., 2020) and the enormous mutational capacity of the causative virus (CCHF virus [CCHFV]; Bente et al., 2013). The virus is mainly transmitted by tick bites and a high proportion of infected people can cope with the infection without clinical manifestations, leading to an important but currently limited impact. Even so, CCHFV is widely distributed in the African, European and Asian continents (Bente et al., 2013), and certain conditions seem to be favouring the emergence of human cases. In the Iberian Peninsula, the virus appears to be endemically established (Cuadrado‐Matías et al., 2022; Moraga‐Fernández et al., 2021). Nevertheless, it is not until the end of the 20th century (1985) that exposure to CCHFV—first described in 1944 (Hoogstraal, 1979)—was reported in Portugal (Filipe et al., 1985). In 2010, CCHFV was detected in *Hyalomma lusitanicum* ticks collected on red deer (*Cervus elaphus*) in western Spain. In 2016, the first human case of CCHF was notified (Cajimat et al., 2017; Negredo et al., 2017). Ten cases with three deaths were notified until 2021 (Sánchez‐Seco et al., 2022) and two additional cases (one lethal) were documented in summer 2022 (ECDC, 2022a). Attention paid to *Hyalomma* spp. ticks in Europe was anecdotal before CCHF human cases were notified (Barandika et al., 2011; Ruiz‐Fons et al., 2006). From then on, *Hyalomma* spp. dynamics was studied in greater depth (Spengler & Estrada‐Peña 2018; Valcárcel et al., 2020). ECDC regularly updates the distribution of European tick species, currently including *H. lusitanicum* (ECDC, 2022b). To date the relationship between *H. lusitanicum* abundance at fine spatiotemporal scales and virus transmission remains unexplored, leaving an important gap in the knowledge of CCHFV ecology. There is no effective vaccine to prevent the potentially serious consequences of CCHFV infection for humans, and thus prevention is the only approach to avoid new human cases.

An essential parameter in ecology and biodiversity conservation is population size of living organisms in ecosystems. Estimating population size is required to estimate their contribution to the ecological communities in which they exist and assess the status of their populations for conservation (e.g. Nunney & Elam, 1994) or population control purposes (e.g. Hagen et al., 2018; Shea, 1998). In epidemiology, knowing the size of the epidemiological population under study is also essential to make inferences about diseases or pathogens in the population (Walton et al., 2016). Species abundance data are generally limited in space and time, especially invertebrates. Only in cases where invertebrates are of conservation interest (Micó et al., 2013), or relevant as pests (Ørsted et al., 2021), or if they are vectors of health‐threatening pathogens (Jaenson et al., 2012), is there interest in promoting research on their spatial distribution and population dynamics. The emergence of CCHF in the Palaearctic and, more specifically, in the European Mediterranean (Estrada‐Peña et al., 2013), shows the need to better understand the ecology and population dynamics of *Hyalomma* spp. ticks for disease prevention.

We recently demonstrated the relevance of the main *H. lusitanicum* host in Iberia, the red deer, to map CCHFV transmission risk (Cuadrado‐Matías et al., 2022). However, most infectious processes are dynamic and linked to the wide variety of environmental and population determinants that modulate the inter‐ and intra‐specific interactions between animal species, or with pathogen vectors (Casades‐Martí et al., 2020; González‐Barrio, Almería, et al., 2015). The rate of pathogen transmission is thus variable in time and space. Being a vector‐borne pathogen for a limited number of their hosts (primarily humans) and able to replicate in many animal species, CCHFV is a successful pathogen. Understanding the causes of variation in transmission risk is paramount to prevent CCHF cases. This study seeks to understand the relationship between the small spatial scale dynamics of *H. lusitanicum* and the exposure of its hosts to CCHFV to better predict virus transmission risks and inform health authorities for more efficient preventive actions.

## 2 MATERIAL AND METHODS

### 2.1 Study area

**FIGURE 1:**
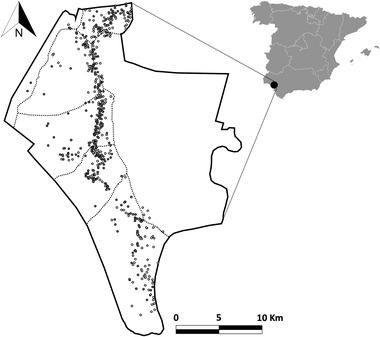
Location of Doñana National Park in peninsular Spain (right) and the exact location of red deer (white dots) and Eurasian wild boar (grey dots) at the sampling time. The boundaries of the five livestock management areas in which the Park is divided to its West are shown (dotted lines)

The study was carried out in Doñana National Park (DNP), in the southwest of peninsular Spain (37°01′N 6°26′W; Figure 1). Doñana is mainly a marshland with large areas of Mediterranean scrubland, pine forests, dunes and sand beaches and a biodiversity‐rich ecosystem. Doñana is a biodiversity hotspot and one of the most important stopover sites for birds migrating annually between Europe and Africa (Rendón et al., 2008). Besides being an essential area for emblematic Iberian endemic species, DNP presents abundant populations of wild ungulates (red deer, fallow deer—*Dama dama*—and Eurasian wild boar—*Sus scrofa*; Barroso et al., 2020) and a limited presence of domestic animals (cattle, sheep and horses). There are no predators for large ungulates in DNP, which has led their populations to grow disproportionately (Barasona et al., 2021). Wild ungulate population control is carried out by the park's environment agents annually through driven hunts. DNP is divided into a series of livestock management units (LMU) whose boundaries are fitted with livestock fencing that does not prevent wildlife movements (see Jiménez‐Ruiz et al., 2022).

DNP is a hotspot for the introduction of infectious agents carried by birds migrating between sub‐Saharan Africa and western Europe. Larvae of abundant exophilic ticks inhabiting north and central Africa, mainly of the genus *Hyalomma*, parasitize migratory birds and travel long distances on them (Palomar et al., 2013). Transported ticks can carry numerous infectious agents (Cajimat et al., 2017; Negredo et al., 2019). Being an essential western Mediterranean stopover for migratory and resident birds (Rendón et al., 2008), environmentally favourable for *Hyalomma* ticks (Williams et al., 2015) and having an abundant ungulate population that is essential for adult *Hyalomma* spp. ticks, DNP constitutes a very relevant hotspot to study the dynamics of infectious vectorial processes shared between Europe and Africa.

### 2.2 Sampling

Between 2005 and 2020, we attended wild ungulate population control events in DNP. The animals were shot by park rangers without considering animals’ sex, age or health status but not for the purpose of this study, so according to current EU and Spanish animal protection regulations, an ethics committee report was not required for this survey. We surveyed red deer and wild boar per year with a homogeneous spatial distribution across DNP each survey year (Table S1). Ticks were counted and collected immediately after the animals were shot. We recorded animals’ sex and age (from tooth eruption patterns; Saénz de Buruaga et al., 2001) and collected blood. Animals were grouped into (i) yearlings (6–12 months old), (ii) juveniles (12–24 months old) and (iii) adults (>24 months old). Samples were preserved refrigerated (4–8°C) during transport to our laboratories where they were conserved until analyses. The location of each shot ungulate was recorded with portable GPS devices.

Ungulate sampling was performed mainly between October and November (Table S1), but animals were sampled throughout the year during the study period. Each individual was assigned a specific sampling season (2005/2006 to 2019/2020) based on sampling date. Each season was defined as the temporal unit of study and run from the 1st of April of one year to the 31st of March of the following year. This was made considering that the probability of exposure to CCHFV depends on *H. lusitanicum* bites and these are active from early spring to late autumn in southern Spain (Valcárcel et al., 2020).

### 2.3 Serological analyses

No animals under 6 months of age sampled by park rangers were selected for serological study due to the potential interference of the presence of maternally derived antibodies with the serological diagnosis of virus exposure (see Casades‐Martí et al., 2020). Sera were obtained after centrifuging blood samples at 10,000 × *g* for 10 min and preserved frozen at –20°C. The presence of specific antibodies against CCHFV in sera was analysed using a commercial ELISA (ID Screen^®^ CCHF Double Antigen Multi‐Species, IDvet, France; Sas et al., 2018) following the manufacturer's indications.

In this study, greater importance was given to yearling specimens as only in this age class does the presence of antibodies to CCHFV indicate that infection was recent (at least within the first year of the animal's life). In adult animals, the presence of antibodies may be either due to recent exposure or from previous infections because CCHFV antibodies are long‐lasting (Negredo et al., 2021) and the ELISA is highly sensitive (Comtet, 2021). Thus, sampling was slightly biased towards yearlings in relation to their proportion in the population. We did not focus only on yearlings because the risk of exposure to *H. lusitanicum* and CCHFV increases with age (Cuadrado‐Matías et al., 2022; Ruiz‐Fons et al., 2013) and our approach required a representative subsample of the overall population.

### 2.4 Environmental predictors

The ecology of CCHFV is intimately linked with the dynamics of hosts and vectors (Bente et al., 2013), and these are influenced by environmental conditions (Ak et al., 2020; Barandika et al., 2011). Inter‐annual fluctuations in host populations are species dependent. While small mammal and bird populations may experience wide interannual fluctuations at local spatial scales (Fargallo et al., 2009), fluctuations in large mammals usually occur on larger time scales. These fluctuations are, to some extent, conditioned by resource availability, which in Mediterranean environments depend on the rainfall regime. Thus, vector‐borne pathogen transmission dynamics depend on variations in both biotic and abiotic environmental factors.

#### 2.4.1 Wild ungulate population

There may be natural slight spatiotemporal variations in large mammals’ availability for CCHFV vectors that could affect virus transmission dynamics. Wild ungulate abundance at LMU level is monitored annually through observation transects (ICTS; http://icts.ebd.csic.es/en/home; Barroso et al., 2020). Each LMU is allocated (at least) one transect (Barasona et al., 2014). Observations allowed estimating the annual kilometric abundance index (KAI) as the average number of individuals of a species per transect kilometre. We estimated the average KAI for each LMU and study year for red deer and wild boar (Figure S1).

#### 2.4.2 Vector abundance

Ticks were counted on 1965 wild ungulates sampled between 2010 and 2020, collected, and morphologically identified to species level (Estrada‐Peña et al., 2017). Tick developmental stage (larva, nymph or adult) and species were recorded, and these were preserved at –80°C. Four species were predominant, including (1) *Rhipicephalus annulatus*, (2) *H. lusitanicum*, (3) *Ixodes ricinus* and (4) *Rhipicephalus bursa*. *Hyalomma marginatum* was found in a much smaller number. With the premise that *H. lusitanicum* is the main vector for CCHFV in DNP, the individual counts of *H. lusitanicum* (*N* = 20,506 ticks) were used as abundance indicators and modelled with a series of environmental predictors (soil temperature, NDVI, land use, host availability) to project its abundance at a 250 × 250 m spatial resolution throughout DNP (Peralbo‐Moreno et al., 2022). Each wild ungulate whose exact shooting location could be georeferenced was assigned the mean value of *H. lusitanicum* abundance predicted for the set of grids included in a 2‐km‐radius buffer around the animal's location at sampling.

#### 2.4.3 Weather predictors

Exophilic ticks are highly sensitive to changes in abiotic environmental conditions (Sobrino et al., 2012). The spatiotemporal scales at which weather conditions influence host and tick abundance and activity vary. We estimated weather conditions using data from the only weather station in DNP (36°59.3ʹN 6°26.6ʹW). Cumulative rainfall values were estimated as indicators of water availability for vegetation growth, vertebrate animals and invertebrates at different periods of the year according to the expected activity of *H. lusitanicum* in Spain (Valcárcel et al., 2020). Thus, accumulated rainfall during the agro‐hydrological year (September 1 to August 31), accumulated rainfall through winter and spring (December–May), spring accumulated rainfall (March–May) and summer accumulated rainfall (June–August) were estimated (Figure S2). Similarly, daily temperature data recorded by the weather station were used to estimate the average winter, spring and summer temperatures (Figure S3).

### 2.5 Data analyses

The data were thoroughly checked to rule out any potential interference in statistical modelling (Zuur et al., 2010). The variability of measurement scales among continuous predictors was reduced by subtracting the mean of each predictor from its values and dividing the result by the standard deviation. We built up the Pearson correlation plot with continuous predictors using the R ‘corrplot’ package (Wei & Simko, 2017), and any collinearity (*r* ≥ |.7|) was removed by selecting the predictors that better explained the variance of the dependent variable. Model parameterization was performed by binary logistic regression using the R packages ‘stats’ and ‘lme4’ (Bates et al., 2015; R Core Team, 2012). To model the influence of *H. lusitanicum* abundance on CCHFV exposure risk, we included tick abundance and ungulate species (red deer vs. wild boar). In this model, we used data from those animals that were georeferenced at sampling (*N* = 807). To estimate the determinants of virus exposure dynamics, we built a generalized linear mixed‐effect model with virus exposure (positive vs. negative) as the response variable and included the selected predictors (Table 1). The sampling season (2005/2006 to 2019/2020) was introduced as a random variable to control for the temporal structure of our sampling design. For this model, we used all available cases (*N* = 980). The process to select the best‐fit model was carried out by building all possible models, ranking them according to their Akaike information criterion (AIC), and calculating the average model with models showing an AIC difference with the best‐fit model (ΔAICc) of less than two units (Table S2); we used the R package ‘MuMIn’ (Barton, 2009). For each model set, we analysed multicollinearity effects by estimating the variance inflation factor (vif) for model parameters (Fox & Weisberg, 2019). A final step in the analysis was to estimate the relative contribution of each model parameter to the variation in the probability of exposure to CCHFV. For this, we partitioned the coefficient of determination *R* ^2^ of the predictors included in the best model by estimating the semi‐partial (part) *R* ^2^ and the structural coefficients with the R package ‘partR2’ (Stoffel et al., 2021). We thus studied the specific contribution of each model predictor to explain the variance of the model. The modelling process was repeated only for yearlings (*N* = 206) to estimate the determinants of the annual CCHFV incidence.

**TABLE 1:** Whole set of predictors considered to be relevant drivers of Crimean–Congo haemorrhagic fever virus transmission grouped within vector, host and weather predictor groups

Predictor group	Predictor	Description	Type and values
Vector	**hlab**	*Hyalomma lusitanicum* abundance (average predicted no. of ticks)	Continuous (0.1–49.4)
Host	**spp**	Host species (Red deer or wild boar)	Categorical (2 classes)
	**dkai**	Red deer kilometric abundance index (average no. of red deer per kilometre)	Continuous (0.3–14.3)
	**wbkai**	Wild boar kilometric abundance index (average no. of wild boar per kilometre)	Continuous (0.0–2.0)
Weather	**ap**	Accumulated rainfall (mm) along the agro‐hydrological year	Continuous (173.0–784.2)
	wsp	Accumulated rainfall (mm) over winter and spring months (December–May)	Continuous (87.4–688.2)
	sp	Accumulated rainfall (mm) along spring months (March–May)	Continuous (28.5–305.3)
	**smp**	Accumulated rainfall (mm) in summer months (June–August)	Continuous (0.0–61.3)
	**wt**	Average temperature (°C) of winter months (December–February)	Continuous (8.1–12.3)
	**st**	Average temperature (°C) of spring months (March–May)	Continuous (15.2–18.0)
	**smt**	Average temperature (°C) of summer months (June–August)	Continuous (22.8–25.5)

*Note*: The predictors that were selected after data descriptive analysis are bold marked.

## 3 RESULTS

**FIGURE 2:**
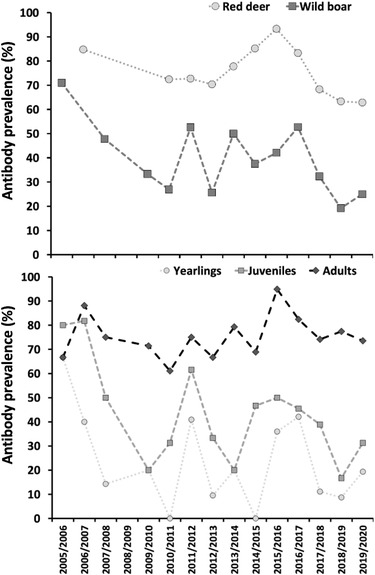
Charts displaying the comparative dynamics of Crimean–Congo haemorrhagic fever virus transmission throughout host species (top chart) and host age class (bottom chart)

The results showed a medium‐to‐high CCHFV exposure prevalence (59.7%; 95% confidence interval [95%_CI_]: 56.6–62.8). Exposure in red deer (76.1%; 95%_CI_: 72.2–79.7) was markedly higher than in wild boar (40.6%; 95%_CI_: 36.1–45.3). Slight variation in annual seroprevalence was observed in red deer, with annual values ranging from 62.9% to 93.3%. The variation was somewhat more accentuated in wild boar, with annual seroprevalence ranging from 19.2% to 71.0% (Table 2). Despite the differences, the temporal pattern of change in annual population seroprevalence was similar for both species (Figure 2), and that displayed a slight negative trend within the time series. The age‐associated increasing exposure pattern was also evident in this study, with yearlings displaying the lowest proportion of exposure to the virus (23.8%; 95%_CI_: 18.2–30.2) when compared to juveniles (42.9%; 95%_CI_: 35.7–50.4) and adults (77.8%; 95%_CI_: 74.1–81.2). The exposure dynamics patterns of yearlings and juveniles were highly similar (Table 3), whereas the temporal pattern in adults was less pronounced and stable within a high seroprevalence (Figure 2). There was a marked variation in the interannual incidence of new cases of CCHFV infection according to observations in yearlings. We observed strong changes between contiguous seasons that in some cases reached a >30% decrease in just one year.

**TABLE 2:** Results of the serological survey for Crimean–Congo haemorrhagic fever virus antibodies in wild ungulates of Doñana National Park throughout ungulate species

	Red deer			Wild boar		
Survey season	pos	*n*	SP	pos	*n*	SP
2005/2006	ns	ns	–	22	31	71.0
2006/2007	78	92	84.8	ns	ns	–
2007/2008	ns	ns	–	11	23	47.8
2008/2009	ns	ns	–	ns	ns	–
2009/2010	ns	ns	–	9	27	33.3
2010/2011	21	29	72.4	7	26	26.9
2011/2012	24	33	72.7	20	38	52.6
2012/2013	19	27	70.4	10	39	25.6
2013/2014	7	9	77.8	20	40	50.0
2014/2015	23	27	85.2	15	40	37.5
2015/2016	56	60	93.3	16	38	42.1
2016/2017	50	60	83.3	29	55	52.7
2017/2018	41	60	68.3	11	34	32.4
2018/2019	38	60	63.3	5	26	19.2
2019/2020	44	70	62.9	9	36	25.0
Total	401	527	76.1	184	453	40.6

Abbreviations: *n*, no. of analysed samples; ns, not surveyed; pos, no. of ELISA‐positive samples; SP, CCHFV seroprevalence (%).

**TABLE 3:** Results of the serological survey for Crimean–Congo haemorrhagic fever virus antibodies in wild ungulates of Doñana National Park throughout survey season and individuals’ age class

	Yearlings			Juveniles			Adults			All		
Survey season	pos	*n*	SP	pos	*n*	SP	pos	*n*	SP	pos	*n*	SP
2005/2006	4	6	66.7	8	10	80.0	10	15	66.7	22	31	71.0
2006/2007	2	5	40.0	9	11	81.8	67	76	88.2	78	92	84.8
2007/2008	1	7	14.3	4	8	50.0	6	8	75.0	11	23	47.8
2008/2009	ns	ns	—	ns	ns	—	ns	ns	—	ns	Ns	—
2009/2010	2	10	20.0	2	10	20.0	5	7	71.4	9	27	33.3
2010/2011	0	2	0.0	5	16	31.3	22	36	61.1	28	55^a^	50.9
2011/2012	9	22	40.9	8	13	61.5	27	36	75.0	44	71	62.0
2012/2013	2	21	9.5	3	9	33.3	24	36	66.7	29	66	43.9
2013/2014	2	10	20.0	2	10	20.0	23	29	79.3	27	49	55.1
2014/2015	0	7	0.0	7	15	46.7	31	45	68.9	38	67	56.7
2015/2016	9	25	36.0	7	14	50.0	56	59	94.9	72	98	73.5
2016/2017	8	19	42.1	10	22	45.5	61	74	82.4	79	115	68.7
2017/2018	2	18	11.1	7	18	38.9	43	58	74.1	52	94	55.3
2018/2019	2	23	8.7	2	12	16.7	24	31	77.4	43	86^b^	50.0
2019/2020	6	31	19.4	5	16	31.3	39	53	73.6	53	106^c^	50.0
Total	49	206	23.8	79	184	42.9	438	563	77.8	585	980	59.7

Abbreviations: *n*, no. of samples; ns, not surveyed; Pos, no. of ELISA‐positive samples; SP, CCHFV seroprevalence (%).

Animals whose age could not be estimated (*n* = 27) were added:

^a^ one animal (one positive);

^b^ 20 animals (15 positive);

^c^ six animals (three positive).

**FIGURE 3:**
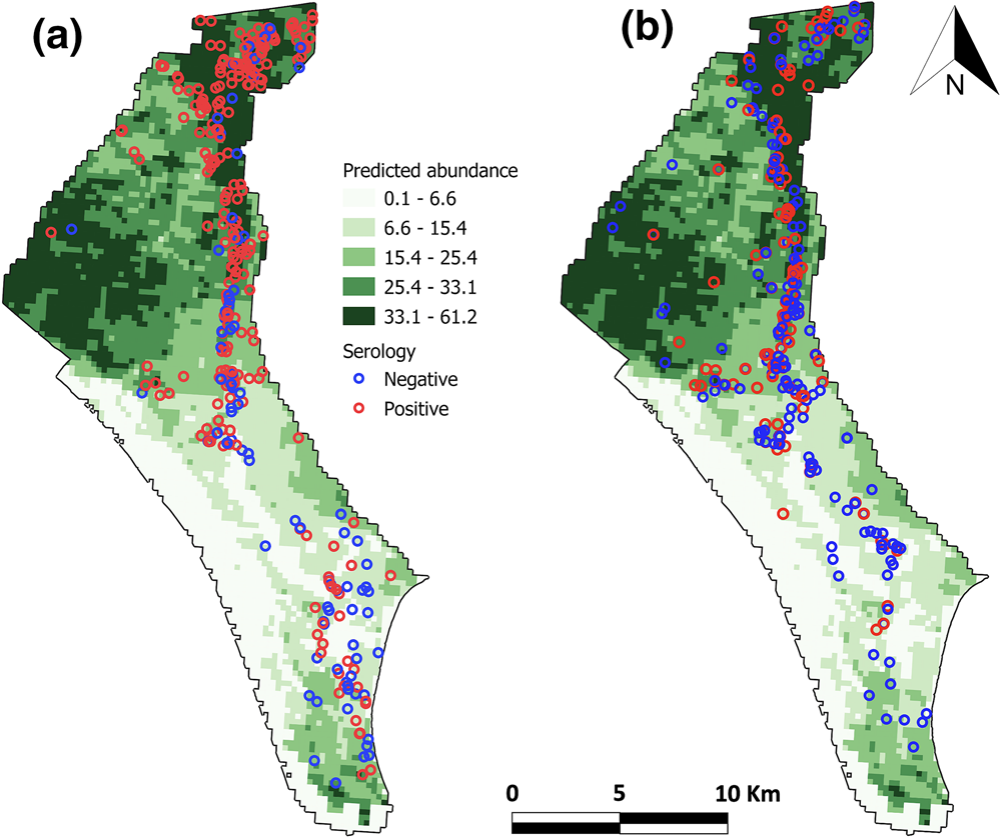
Spatial relationship between predicted *Hyalomma lusitanicum* abundance (average number of ticks per individual wild ungulate host) at UTM 250 × 250 m and the patterns of exposure to Crimean–Congo haemorrhagic fever virus of red deer (a) and Eurasian wild boar (b)

We found a very robust relationship between the spatial distribution of predicted *H. lusitanicum* abundance and the risk of exposure to CCHFV (Table 4; Figure S4). This spatial pattern was similar for red deer and wild boar, with the northern part of DNP as the area of highest exposure risk (Figure 3). We observed that the clear contrast in CCHFV seroprevalence between red deer and wild boar was evident and the difference was statistically significant (Table 5; Figure S5). However, the yearling model showed no host effect on virus exposure risk (Table 5; Figure S6). At the scale of the study, we observed a marked effect of wild ungulate abundance, with higher risk at higher deer abundance and with a limited but negative influence of wild boar abundance (Table 5; Figure S5). The results of the annual incidence modelling also indicated a significant positive effect of deer abundance on exposure risk (Figure S6), confirming it as an important risk factor. Weather predictors were overall less important in determining exposure risk, but the model results show their risk‐modulating capacity. The influence of rainfall on exposure risk was contrasted, with an overall lower risk in rainy years, but with a positive effect of the accumulated precipitation during the summer drought. We observed a positive risk‐modulating capacity of summer temperature which, although with a lower effect when compared to host factors, also appears as a relevant risk factor for the annual risk of exposure to CCHFV. The variance decomposition of the model into its predictor components corroborated the results of the prevalence and incidence models (Figures S7 and S8). Predictors grouped as vector and virus host related (host species and red deer abundance) were shown to be the most relevant risk factors for CCHFV exposure in DNP, explaining very significant proportions of the model variance. Weather predictors showed a less relevant weight as risk factors.

**TABLE 4:** Output of the generalized linear model performed to analyse the timeless but spatially explicit influence of *Hyalomma lusitanicum* abundance on the risk of exposure of wild ungulates to Crimean–Congo haemorrhagic fever virus

Predictor	Estimate	*SE*	*z*	*p*
Intercept	1.1134	0.1163	9.572	***
spp	–1.5115	0.1589	–9.513	***
hlab	0.5594	0.0802	6.974	***

*Note*: The table shows predictors (see Table 1), their estimates and associated standard errors (SE), the statistic (*z*) and the *p*‐value.

*p < .05; **p < .01; ***p < .001.

**TABLE 5:** Output of the average generalized linear mixed models selected to identify the drivers of Crimean–Congo haemorrhagic fever virus transmission dynamics in the general wild ungulate population and in the yearlings group

Model database	Predictor	Estimate	*SE*	*z*	*p*
General	Intercept	1.3499	0.1539	8.762	***
	spp	−1.7562	0.1685	10.409	***
	dkai	0.5215	0.0956	5.451	***
	wbkai	−0.1817	0.0779	2.327	*
	ap	−0.4445	0.1507	2.946	**
	smp	0.3795	0.1124	3.372	***
	smt	0.4304	0.1339	3.211	**
	wt	−0.0769	0.1294	0.594	ns
Yearlings	Intercept	−1.2145	0.3399	3.557	***
	dkai	0.4003	0.1824	2.181	*
	smt	0.3942	0.2960	1.327	ns
	spp	−0.0953	0.2801	0.339	ns
	ap	−0.0361	0.1245	0.289	ns
	wt	−0.0274	0.1176	0.232	ns

*Note*: The table shows predictors, their estimates and associated standard errors (SE), the statistic (*z*), and the *p*‐value. Predictors shown as abbreviations according to Table 1.

*p < .05; **p < .01; ***p < .001.

## 4 DISCUSSION

Some of the first questions to be solved after the emergence of human cases of CCHF in Spain and the resulting alarm, such as the distribution and presence of CCHFV, have been resolved (Cuadrado‐Matías et al., 2022; Frías et al., 2022; Monsalve Arteaga et al., 2021; Moraga‐Fernández et al., 2021; Sánchez‐Seco et al., 2022). However, many aspects of the ecology of the virus remain unresolved and they are essential to prevent CCHF cases. Identifying risk indicators could be of great relevance to improve prevention schemes beyond mapping risk zones, as well as identifying the robustness of these indicators at varying temporal and spatial scales. In this work, we identify two very relevant factors for the spatial and temporal transmission patterns of CCHFV in an enzootic area: (1) the abundance of *H. lusitanicum* at small spatial scales and (2) the host community structure at local spatial and annual temporal scales.

### 4.1 Methodological issues

The selection of an animal study model proved to be a good choice for detecting PCR‐positive ticks (Estrada‐Peña et al., 2012; Moraga‐Fernández et al., 2021; Sánchez‐Seco et al., 2022) and mapping exposure risk (Cuadrado‐Matías et al., 2022). The selection of the study area, where the virus was detected at high prevalence in ticks collected from wild ungulates (The authors, unpublished), also proved to be a good choice. An important bias in studying pathogen transmission dynamics using the frequency of antibody presence is the time that those antibodies remain detectable after exposure. If we assume that the average life of CCHFV antibodies is long (Bartolini et al., 2019; Negredo et al., 2021), there is a high level of imprecision in determining when exposure occurred with our survey approach to properly understand virus transmission dynamics. To counteract this imprecision, we assumed that those animals aged 6 months to 1 year may have contacted the virus only during their first year of life, thus constituting an annual incidence value indicative of the likelihood of virus transmission. Further, antibodies can be transmitted as passive immunity from mother to offspring (see González‐Barrio, Fernández‐de‐Mera, et al., 2015). To rule out the detection of maternally derived antibodies, no animals younger than 6 months old were included in the study. We therefore modelled the probability of exposure to the virus in the general population and, specifically, in yearlings.

Modelling *H. lusitanicum* burdens on hosts on an annual basis could yield highly imprecise predictions due to limited sample size, so its abundance model was represented with spatial but not temporal accuracy. Therefore, we separately analysed the timeless influence of *H. lusitanicum* spatial abundance and the temporal influence of additional environmental predictors. We were thus unable to explore whether the potential temporal variation in the spatial patterns of vector abundance could influence the exposure to CCHFV. However, we may assume that environmental favourability at the landscape level is sustained over time even when weather modulates vector annual abundance. We would expect that more environmentally favourable areas for *H. lusitanicum* would always yield higher abundance than less favourable areas, at least at the spatial scale of DNP, and that is what we observed (Peralbo‐Moreno et al., 2022).

### 4.2 Exposure risk determinants

The structure of the community of *H. lusitanicum* hosts is an important risk factor for CCHFV transmission. Previous studies already pointed out high *H. lusitanicum* burden on red deer in Iberia (Ruiz‐Fons et al., 2013), also in DNP (Peralbo‐Moreno et al., 2022). Wild boar host *H. lusitanicum* (Ruiz‐Fons et al., 2006), but with much lower burdens than deer at similar parasitization prevalence. This fact may explain the difference in exposure risk observed between wild boar and red deer. This is probably related to several anatomical, physiological and behavioural trait variations such as the smaller size, the lower sexual dimorphism or the habit of wild boar of fighting against ectoparasites through mud baths (Fernandez‐Llario et al., 2005). Wild boar could also show variation in space use (habitat preferences) compared to deer, which could also have an effect in the frequency by which they use *H. lusitanicum* favourable areas.

The models that best explain variation in the incidence of new infections clearly point out the weight of vector‐ and virus host‐related factors. However, and from a practical point of view, we should consider that it is not advisable to dissociate these effects from the influences of abiotic climatic factors that have also been shown to be relevant drivers of CCHFV transmission risk. The concordance between factors modulating the abundance of the main CCHFV vector in DNP (Peralbo‐Moreno et al., 2022) and those modulating the risk of exposure to the virus is striking, but in a way completely logical. This clearly shows the impossibility of separating vector, host and virus, and especially corroborating the role of *H. lusitanicum* in the Iberian Peninsula. At present, it seems unlikely that *H. lusitanicum* is spreading outside its main areas of distribution in the Iberian Peninsula and North Africa. This is probably not because environmental favourable conditions for the species are not improving in areas where it is not present, as it is happening for *H. marginatum* (Bah et al., 2022) and *Hyalomma scupense* (Grech‐Angelini et al., 2016), but because its movements are probably closely linked to the natural movements of its main hosts that only occasionally include birds. However, the species appears in low abundance in areas where it had not been documented, perhaps associated with notable changes in the population dynamics of its hosts (Castillo‐Contreras et al., 2021). A notable change in the favourable environmental conditions for *H. lusitanicum* and other exophilic ticks has been, apart from climate change, the unprecedented expansion of wild ungulate populations in the northern hemisphere, not only in spatial distribution but also in densities (Valente et al., 2020). The intimate relationships that hematophagous arthropods establish with their hosts make them highly host dependent. *Hyalomma lusitanicum* parasitize mainly ungulates, so its abundance is highly dependent of ungulate availability (e.g. see Ruiz‐Fons & Gilbert, 2010 for *I. ricinus*). If in parallel to wild ungulate growth there is no increase in the control of their parasites, these may have increased in favourable areas (e.g. Acevedo et al., 2010) to the point that their pathogens are experiencing spill over events, even causing unexpected cases (ECDC, 2022a). This hypothesis seems plausible as an explanation for the emergence of CCHF in Spain within an enzootic scenario. Thus, the recommendation to reduce the risk of CCHFV infection would be reducing wild ungulate density. However, this a priori simple suggestion needs from a robust evidence of the role played by domestic and wild animals in CCHFV ecology, in addition to finding out to what extent CCHFV has adapted or may be trying to adapt to other vectors (Moraga‐Fernández et al., 2021).

## 5 CONCLUSIONS

Despite the different measures of incidence and prevalence in yearlings and the total population, respectively, we note that determinants of inter‐annual variation in virus exposure are also predominantly host related. This perhaps points out that although less appropriate, population seroprevalence may be a good temporal parameter over long time scales to study the determinants of virus transmission risk. This is especially relevant for studies to be carried out in areas where, unlike our specific case, sampling of wild ungulates is performed in hunting events where the number of yearlings shot is usually very low (see Casades‐Martí et al., 2020 for details about specific age‐class shooting in hunting events). However, to monitor temporal variations in pathogen exposure, it is still recommendable including yearlings in surveys (Cuadrado‐Matías et al., 2022; González‐Barrio, Fernández‐de‐Mera, et al., 2015).

This work provides relevant insights into the transmission dynamics of CCHFV in enzootic scenarios, not only providing important information to reduce the risk of virus transmission by taking actions according to our findings, but also to deepen the understanding of the ecology of CCHFV and its major determinants.

## AUTHOR CONTRIBUTIONS

Francisco Ruiz‐Fons acquired and managed funding. Francisco Ruiz‐Fons and Raúl Cuadrado‐Matías conceived and designed the study and drafted the manuscript. Francisco Ruiz‐Fons, Raúl Cuadrado‐Matías and Gloria Herrero‐García performed the statistical analyses. Sara Baz‐Flores and Alfonso Peralbo‐Moreno performed tick morphological identification and *Hyalomma lusitanicum* abundance models. Raúl Cuadrado‐Matías performed serological analyses. All the authors collected the samples, contributed with data for analyses and revised and agreed the final version of the manuscript.

## CONFLICT OF INTEREST

The authors declare no conflict of interest.

## ETHICS STATEMENT

The study was conducted according to the guidelines of the Declaration of Helsinki.

## Supporting information

Supporting Information

## Data Availability

The data that support the findings of this study are available from the corresponding author upon reasonable request.
