# Supplementary material for: Determinants of Crimean–Congo haemorrhagic fever virus exposure dynamics in Mediterranean environments
Source: Transbound Emerg Dis. 2022 Oct 17;69(6):3571–81. doi: 10.1111/tbed.14720 (PMC10092370; doi:10.1111/tbed.14720)
Supplement: Supplementary file 1 — Supporting Information [file TBED-69-3571-s001.docx]

**Supplementary Figure 1.** Temporal trend (dotted lines) of the Kilometric Abundance Index (KAI) of (a) red deer and (b) wild boar in DNP throughout livestock management areas that are (in a north-to-south gradient; see Figure 1): i) Coto del Rey; ii) Los Sotos; iii) Reserva Biológica de Doñana; iv) El Puntal; and v) Marismillas.


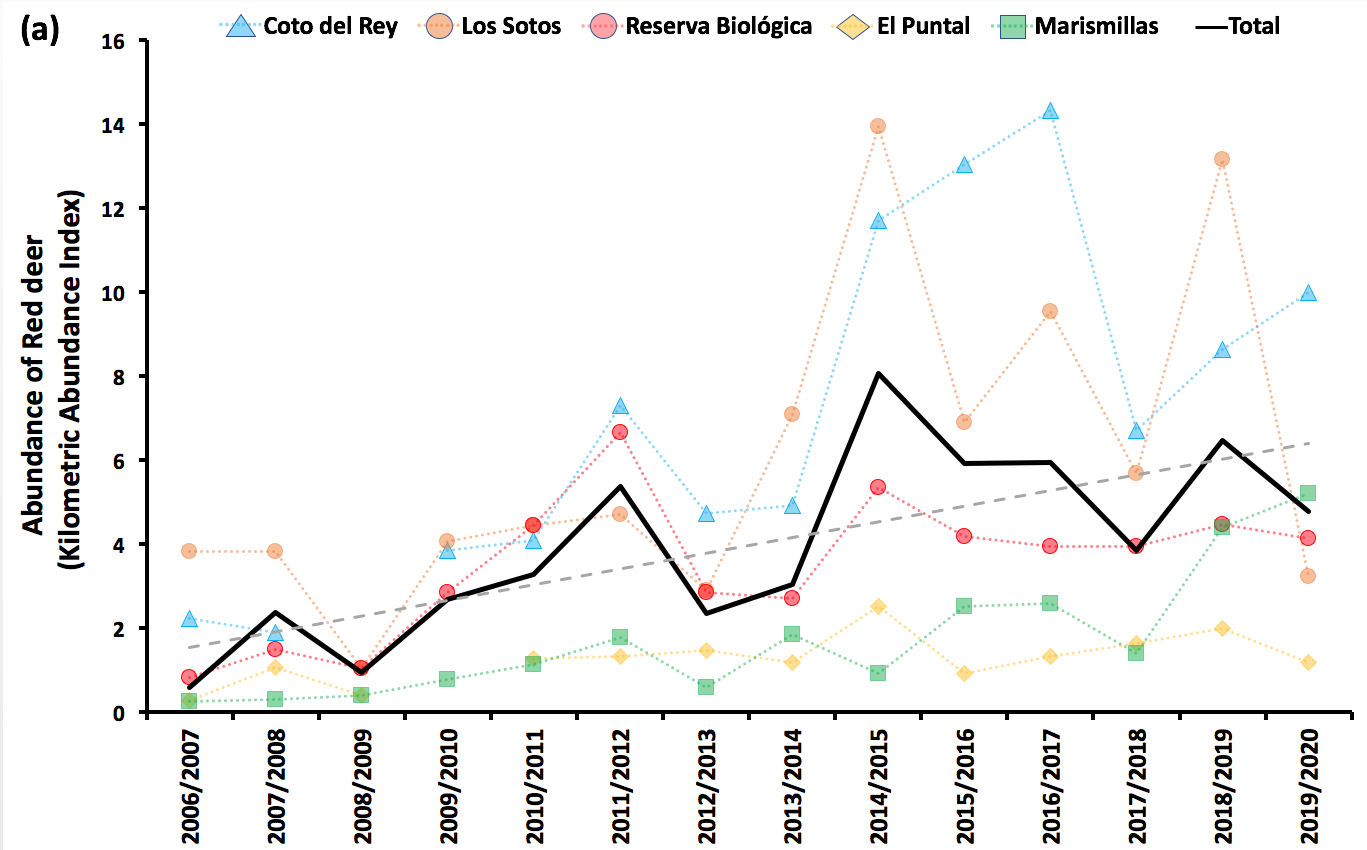


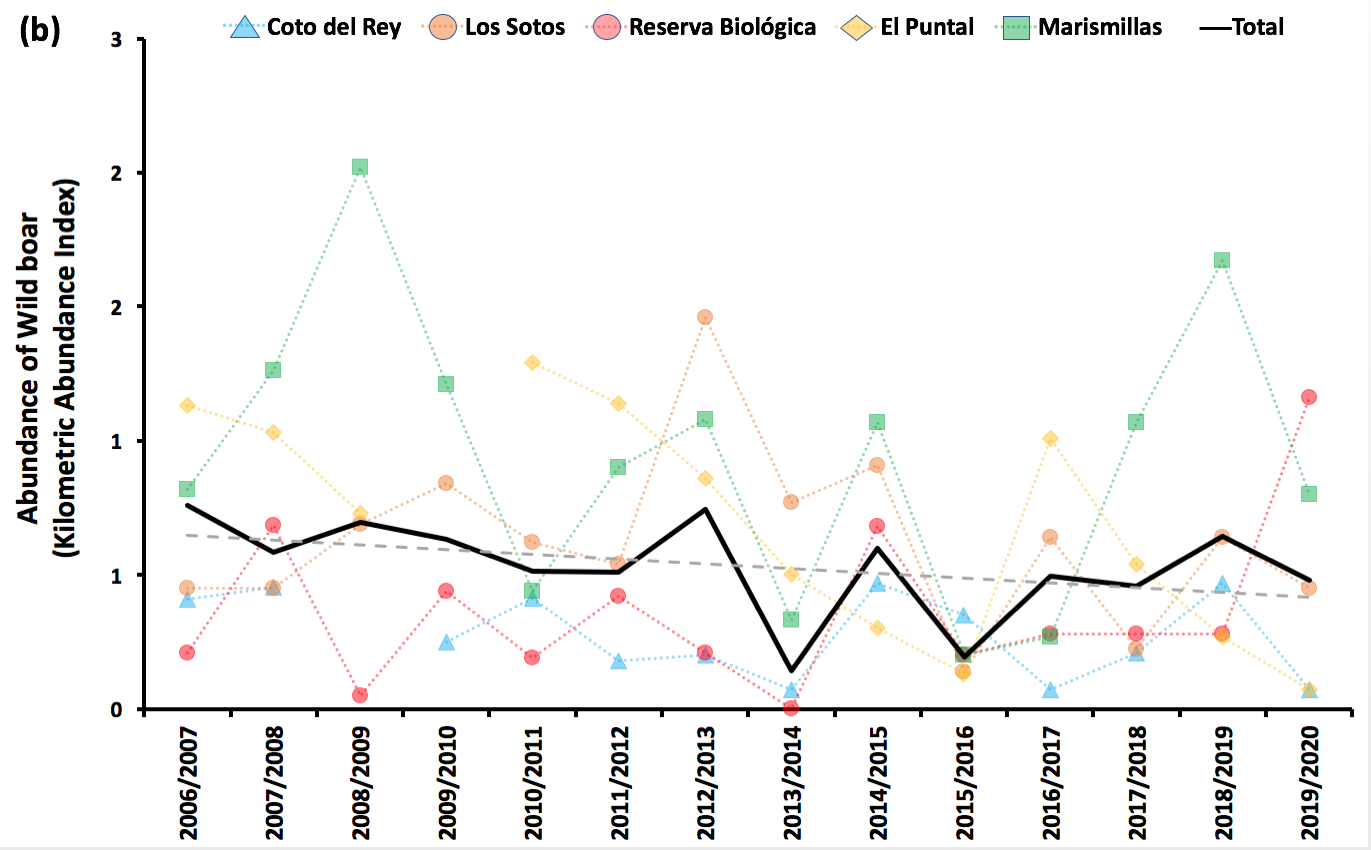


**Supplementary Figure 2.** Chart displaying cumulative precipitation during the agro-hydrological year in a temporal trend from 2005 to 2020. Variables recorded at the DNP weather station.


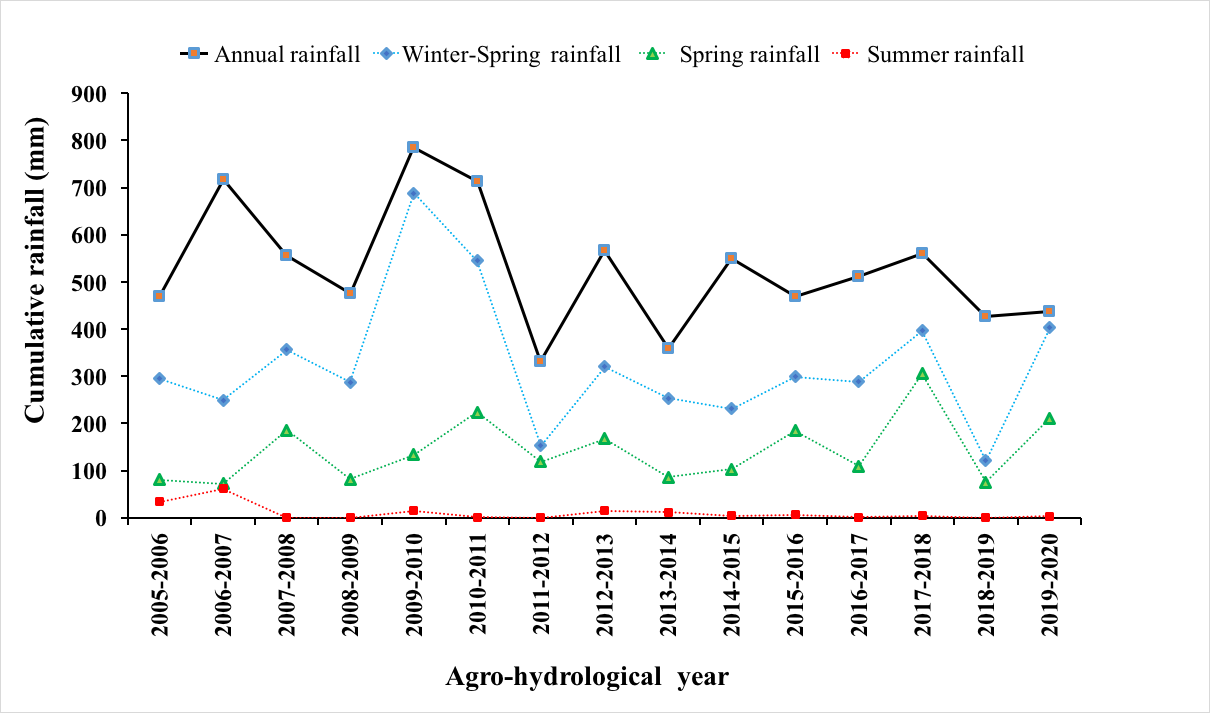


**Supplementary Figure 3.** Variations in average temperatures over the years at DNP. The red line displays the average summer temperatures, in green the average spring temperature and in blue the average winter temperature. The black line represents the average temperature for the total year. Variables recorded at the DNP weather station.


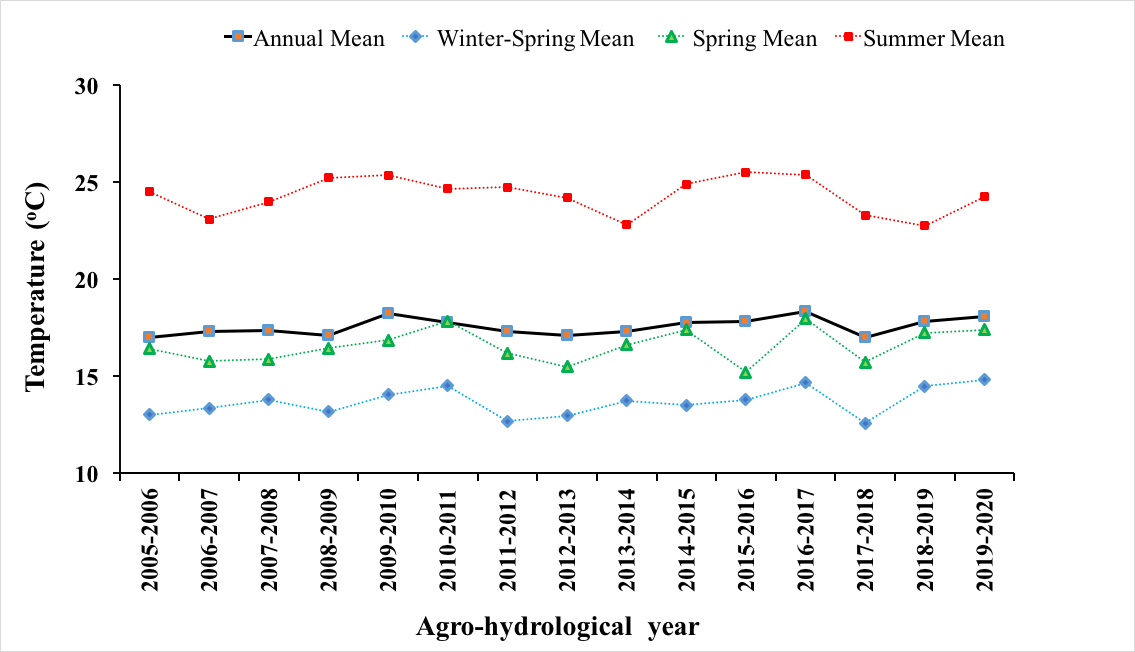


**Supplementary Figure 4.** Output of the generalised linear model performed to explore the influence of *Hyalomma lusitanicum* abundance on the risk of wild ungulated being exposed to CCHFV.


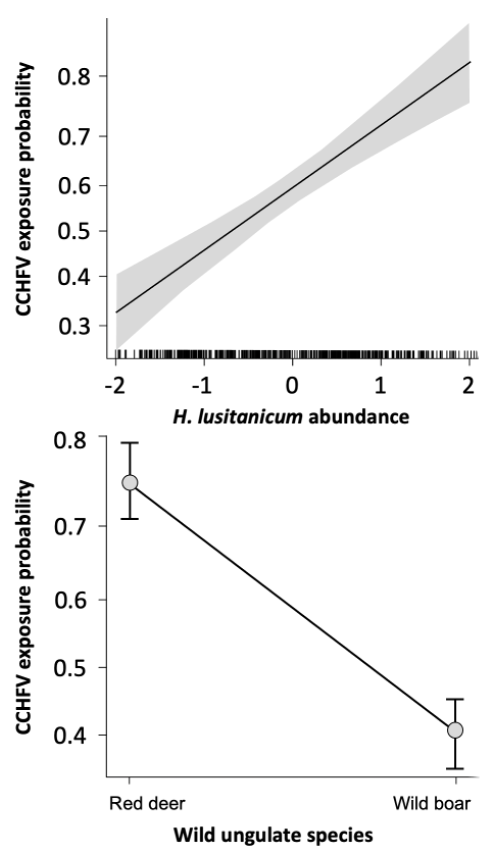


**Supplementary Figure 5.** Model output charts of the general population dynamics transmission model.


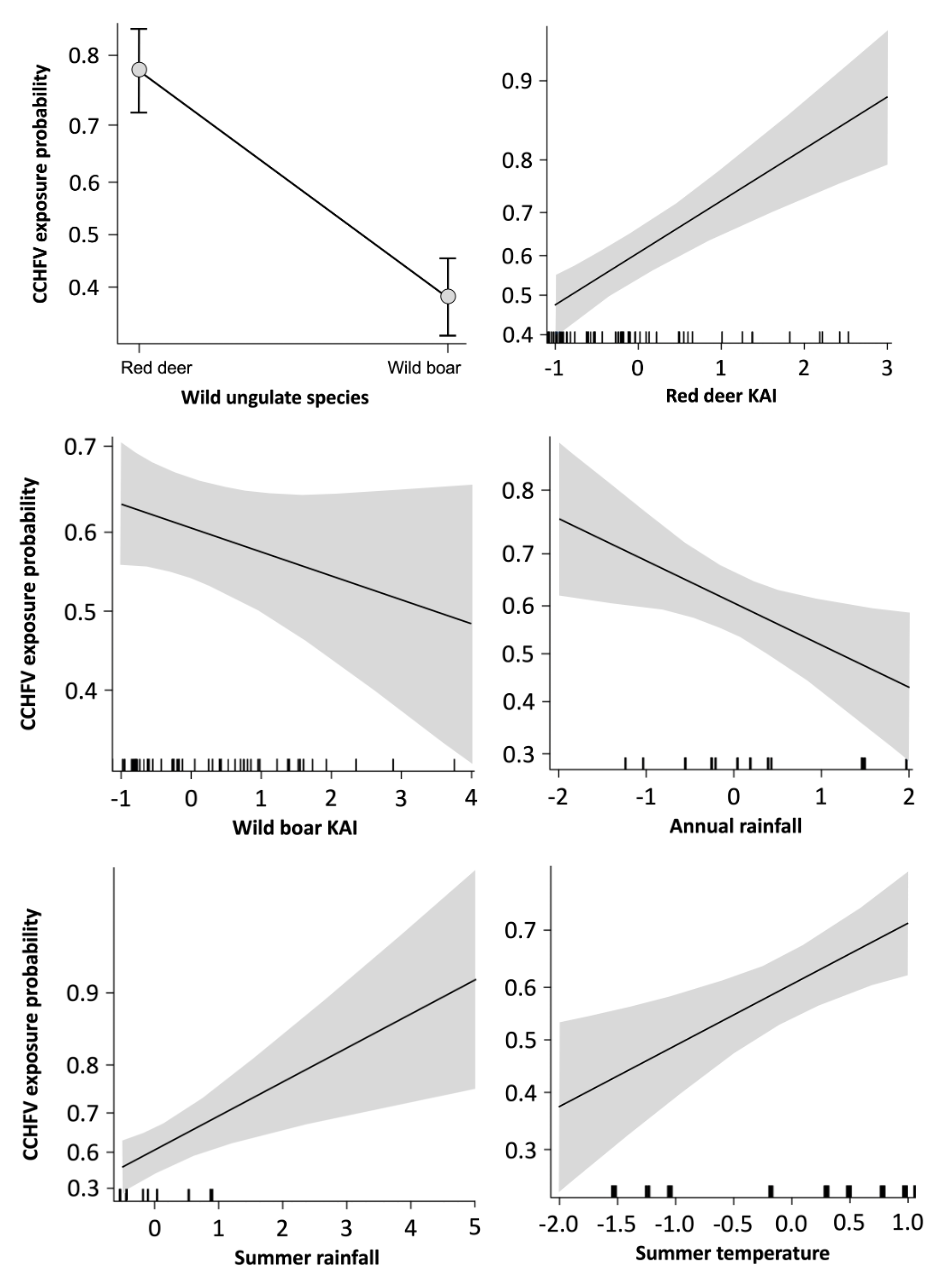


**Supplementary Figure 6.** Model output charts of the yearling population dynamics transmission model.

**
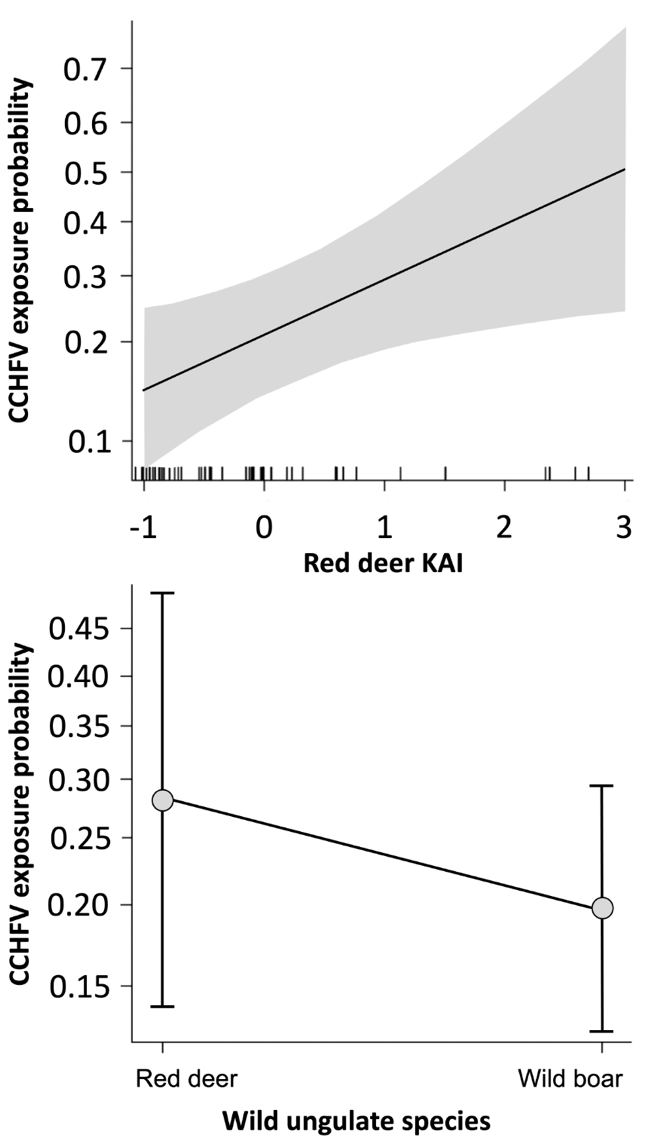
**

**Supplementary Figure 7.** Forestplots for comparison of part R2 for model predictors (A), inclusive R^2^ (B), structure coefficients (C) and beta weights (C) including confidence intervals (CI) for the general population risk model.


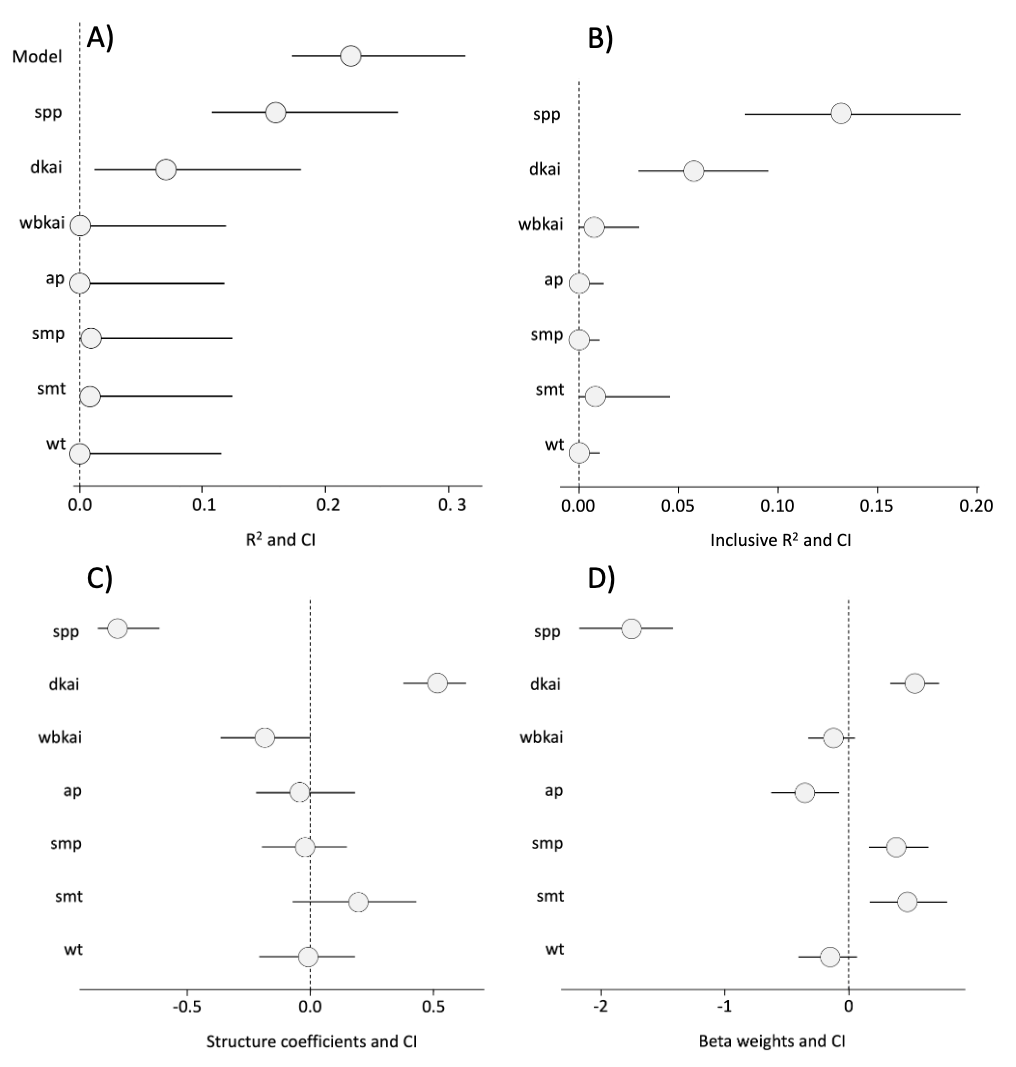


**Supplementary Figure 8.** Comparison of part R2 for model predictors (A), inclusive R2 (B), structure coefficients (C) and beta weights (C) including confidence intervals (CI) for the yearlings’ risk model.


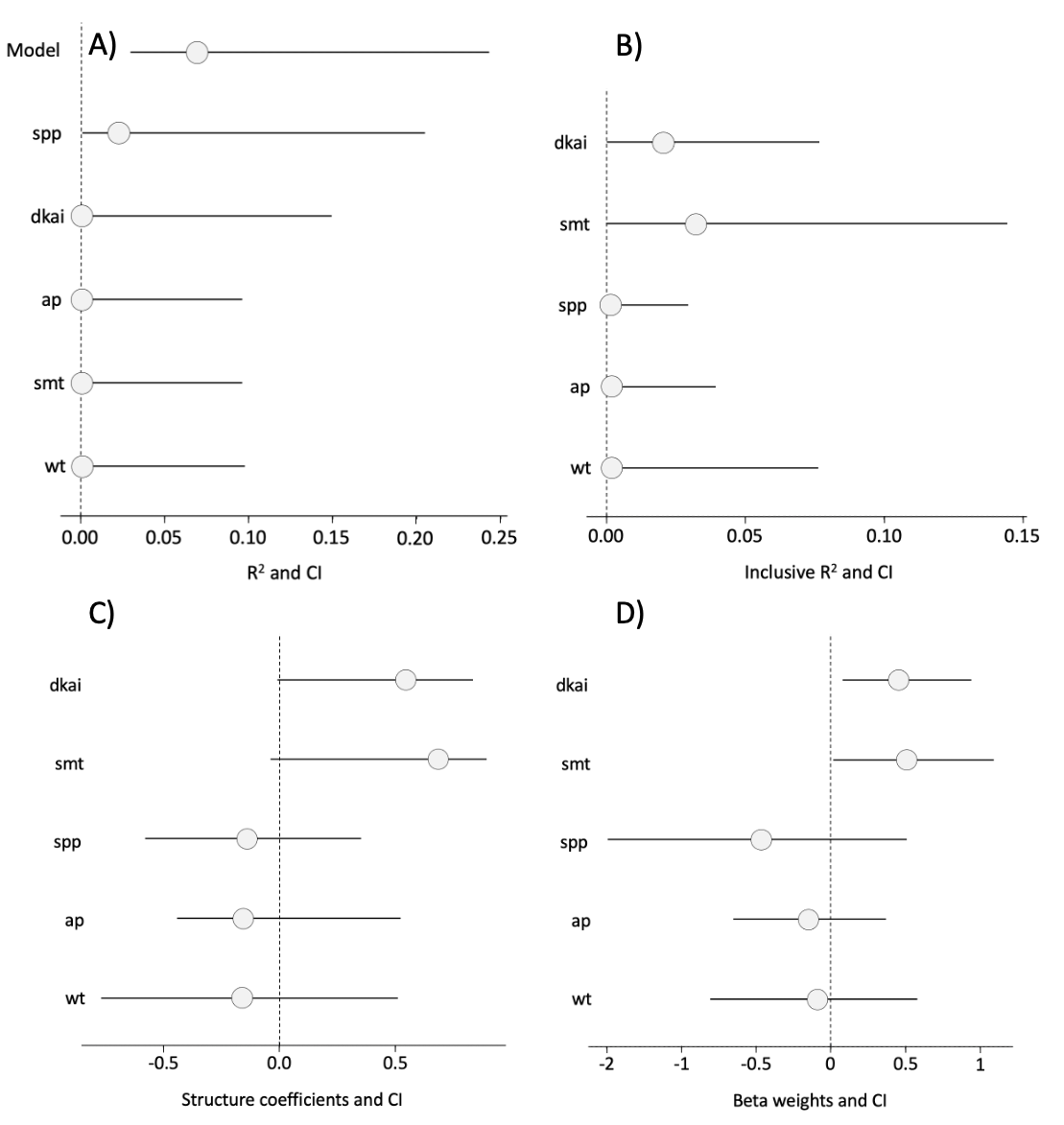


**Supplementary Table 1.** Number of wild ungulates sampled per season and month during the study period.

| **Season** | JAN | FEB | MAR | APR | MAY | JUN | JUL | AUG | SEP | OCT | NOV | DEC |
| --- | --- | --- | --- | --- | --- | --- | --- | --- | --- | --- | --- | --- |
| **2005/2006** | --- | --- | 31 | --- | --- | --- | --- | --- | --- | --- | --- | --- |
| **2006/2007** | 12 | 14 | 6 | 26 | --- | --- | --- | --- | --- | 10 | 12 | 12 |
| **2007/2008** | --- | 23 | --- | --- | --- | --- | --- | --- | --- | --- | --- | --- |
| **2008/2009** | --- | --- | --- | --- | --- | --- | --- | --- | --- | --- | --- | --- |
| **2009/2010** | --- | --- | --- | --- | --- | --- | --- | --- | --- | --- | 27 | --- |
| **2010/2011** | --- | 1 | --- | --- | --- | --- | --- | --- | 36 | 18 | --- | --- |
| **2011/2012** | --- | --- | --- | --- | --- | --- | 6 | 20 | 2 | 8 | 35 | --- |
| **2012/2013** | --- | --- | --- | 10 | 2 | --- | 4 | 2 | --- | 48 | --- | --- |
| **2013/2014** | 1 | --- | --- | --- | --- | 5 | 32 | --- | 2 | 9 | --- | --- |
| **2014/2015** | --- | 2 | --- | --- | --- | --- | 3 | 19 | --- | 2 | 41 | --- |
| **2015/2016** | --- | 1 | --- | --- | --- | 11 | 11 | --- | 10 | 15 | 41 | 9 |
| **2016/2017** | 4 | 2 | 11 | --- | --- | --- | 1 | --- | --- | 57 | 30 | 10 |
| **2017/2018** | 13 | --- | --- | --- | 3 | --- | 2 | --- | --- | 68 | 7 | 1 |
| **2018/2019** | --- | --- | --- | --- | --- | --- | --- | --- | --- | 53 | 33 | --- |
| **2019/2020** | --- | --- | --- | 6 | --- | --- | --- | --- | 9 | 77 | 5 | 9 |
| ***Total*** | *30* | *43* | *48* | *42* | *5* | *16* | *59* | *41* | *59* | *365* | *231* | *41* |

**Supplementary Table 2.** Set of models with ΔAIC<2 selected for model averaging. The coefficient estimates of the predictors included in the models are highlighted in bold.

| **Model** | Δ**AIC / weight** | **Predictors** | | | | | | | |
| --- | --- | --- | --- | --- | --- | --- | --- | --- | --- |
|  |  | **spp** | **dkai** | **wbkai** | **ap** | **smp** | **wt** | **st** | **smt** |
| Gral.1 | 0.00/  0.256 | + | 0.5191 | -0.1793 | -0.4877 | 0.3858 | ns^a^ | ns | 0.4370 |
| Gral.2 | 0.56/  0.194 | + | 0.5246 | -0.1848 | -0.3872 | 0.3711 | -0.1785 | ns | 0.4218 |
| Yearl.1 | 0.00/  0.058 | ns | 0.3864 | ns | ns | ns | ns | ns | 0.4679 |
| Yearl.2 | 0.97/  0.035 | + | 0.4074 | ns | ns | ns | ns | ns | 0.4909 |
| Yearl.3 | 1.14/  0.033 | ns | 0.3800 | ns | ns | ns | ns | ns | ns |
| Yearl.4 | 1.21/  0.031 | ns | 0.4198 | ns | -0.2114 | ns | -0.1868 | ns | 0.4855 |
| Yearl.5 | 1.51/  0.027 | ns | 0.4223 | ns | ns | ns | ns | ns | 0.4798 |

^a^ns: predictors not selected in specific models.
